# Intrahippocampal Injection of 3*α* Diol (a Testosterone Metabolite ) and Indomethacin (3*α*-HSD Blocker), Impair Acquisition of Spatial Learning and Memory in Adult Male Rats 

**Published:** 2013

**Authors:** Somayeh AssadianNarenji, Nasser Naghdi, Shahrbanoo Oryan, Kayhan Azadmanesh

**Affiliations:** a *Department of Biology, Science and Research Branch, Islamic Azad University, Tehran, Iran.*; b * Department of Physiology and Pharmacology, Pasteur Institute of Iran, Tehran 13164, Iran.*; c *Department of Virology, Pasteur Institute of Iran, Tehran 13164, Iran. *

**Keywords:** Androgens, Spatial memory, 3*α *diol, indomethacin, Morris water maze, GABA_A _receptor

## Abstract

Hippocampus is essentially involved in learning and memory processes, and is known to be a target for androgen actions. The high density of the androgen receptors in hippocampus shows that there must be some relationship between androgens and memory. Androgen effects on spatial memory are complex and contradictory. Some evidence suggests a positive correlation between androgens and spatial memory. While some other reports indicated an impairment effect. The present study was conducted to assess the effect of 3*α *diol on spatial discrimination of rats. Adult male rats were bilaterally cannulated into CA1 region of hippocampus and then received 3α diol (0.2, 1, 3 and 6 μg/ 0.5 μL/side), indomethacin (1.5, 3 and 6 μg/ 0.5 μL/side), indomethacin (3 μg/ 0.5 μL/side) + 3α diol (1μg/ 0.5 μL/side), 25-35 min before training in Morris Water Maze task. Our results showed that injection of 3*α *diol (1, 3 and 6 μg/ 0.5 μL/ side) and indomethacin (3 and 6 μg/ 0.5 μL/side) significantly increased the escape latency and traveled distance to find hidden platform. It is concluded that intra CA1 administration of 3α diol and indomethacin could impair spatial learning and memory in acquisition stage. However, intra hippocampal injection of indomethacin plus 3*α *diol could not change spatial learning and memory impairment effect of indomethacin or 3*α *diol in Morris Water Maze task.

## Introduction

Steroid hormones are synthesized in the gonads and reach brain via the blood circulation (1, 2). In addition, the local endogenous synthesis of estrogens and androgens from cholesterol occurs in glial cells, astrocytes and neurons in the central and peripheral nervous system by the mediation of cytochrome P_450_ and non P_450_ enzymes (1-4). Steroids not only affect the sexual behavior responses, but also the ability of the brain to process, store and retrieve sensory information. Neuromodulatory function of steroid hormones have been investigated in the hippocampus, because the hippocampus is attractive as a center of learning and memory (2). Androgens can enhance neural excitability in the hippocampus of male rats and increase dendritic spine density in the CA1 and CA3 regions of the dorsal hippocampus (5). 

Changes in gonadal steroids levels over the time of life, in addition to causing the variations in cognitive function, contribute to neurodegenerative disorder such as Alzheimer’s disease (AD) (6-8). 

The male rat hippocampus is rich in androgen receptors expressing cells (1, 9, 10), so that the much of the work on steroid-induced learning, has focused on the effects of androgens in the hippocampal spatial memory. The literature of androgen effects on spatial memory is complex and contradictory (1, 5, 9-11). Some evidence suggests that androgens can impair memory in animals (12). It has been shown that the injection of testosterone in the CA1 region of hippocampus impaired spatial memory in adult male rats (1, 9, 10, 13-17). Several reports also indicated that chronic treatment with androgens impaired spatial learning and retention of spatial information in young and middle-aged animals (13, 18). In contrast, some studies show a positive correlation between testosterone (T) and its metabolites and spatial ability (19-23). For instance men with lower levels of T due to hypogonadism or aging demonstrate poorer cognitive performance, and these deficits can be reduced by androgen-replacement therapy (21, 24). Also studies in animal models have shown that gonadectomized (GDX) male rodent show poorer performance in spatial learning (25).

One of the complexities in understanding the effects of androgens is that these steroids have very broad spectrum of activity. At least in part, this is because androgens represent substrate for the synthesis of several biologically active metabolites (6, 26). 

Our previous studies showed that testosterone impaired learning and memory (1, 5, 9, 10, 11). An important question is how it may have its effects on spatial memory. Testosterone is readily metabolized in the brain by the 5*α*-reductase enzyme to dihydrotestosterone (DHT), which is subsequently converted by the 3*α *–hydroxysteroid dehydrogenase enzyme (3*α*-HSD) to nonaromatizable metabolite 5*α *–androstan-3*α*, 17*β*- diol (3α-diol) (20, 27, 28). Systematic administration of 3*α*-diol to gonadectomized (GDX) rats enhances cognitive performance in the inhibitory avoidance, place learning and object recognition tasks (28); however, there is no information about the effect of intrahippocampal injection of 3*α*-diol on spatial learning and memory.

Indomethacin is a poorly water-soluble, non-steroidal anti-inflammatory drug (29) anda prostaglandin blocker which powerfully inhibits 3*α*-HSD reduction (30). Indomethacin can act as a 3*α*-HSD inhibitor, then, by blocking testosterone and DHT‘s metabolism to 3*α*-diol, it can affect memory process. Some of studies have shown that indomethacin as 3*α*-HSD blocker may affect on testosterone and 3*α*-diol metabolism in spatial learning and memory procedure (20, 28). In addition, it is well known as a nonselective COX inhibitor. COX (cyclooxygenase) enzymes catalyze the first two committed steps in the biosynthesis of prostanoids (30-33). Indomethacin as a nonselective COX inhibitor, can affect on learning and memory. Several lines of evidence indicate a potential role for COX in the physiological mechanisms underlying memory function. For example, nonspecific COX inhibitors such as indomethacin impair passive avoidance memory in chicks and prevent the learning-induced increase in prostaglandin (PG) release, which occurs 2 h after training (33). 

Because of the contradictory effects of testosterone and its metabolites on spatial learning and memory and also as there is no information about hippocampal effect of 3*α*-diol, in this study we investigated the effect of intrahippocampal administration of 3α-diol on learning and memory in presence or absence of indomethacin (3*α*-HSD blocker) in Morris Water Maze task. 

## Experimental

Male albino Wistar rats (200-250 g) obtained from the Pasteur Institute of Iran were used in experiments. Rats were housed five per cage in large cages before surgery and individually in small cages after surgery, and were maintained at room temperature of 23 ± 2 ºC under standard 12:12 h light-dark cycle with lights on at 07:00 am. Food and water were available *ad libitum*. Experimental procedures were carried out in accordance with the recommendations internationally accepted principles for the use of experimental animals.

*Surgery*

Rats were anesthetized using a ketamineandxylazine (100 mg/kg and 25 mg/kg, IP) and placed in a stereotaxic instrument (Stoelting, USA). Bilateral guide cannulas were implanted in the right and left CA1 and were attached to the skull surface using dental cement jeweler’s screws. Stereotaxic coordinates based on Paxinos and Watson’s Atlas of the rat brain (34) was: anterior-posterior (AP), -3.8 mm from bregma; medial-lateral (ML), ±2.2 mm from midline; and dorsal-ventral (DV), -2.7 mm from the skull surface. 

*Microinjection procedure*

Intracerebral injections were administered through guide cannula (23-gauge) using injection needles (30-gauge) connected by polyethylene tubing to 0.5 μL Hamilton microsyringes. The injection needle was inserted 0.5 mm beyond the tip of the cannula. Then 0.5 μL of vehicle (dimethyl sulfoxide, DMSO) or different doses of 3*α*- diol (Sigma, U.S.A) and indomethacin **(**Daroopakhsh Co. Iran) were injected into each side of CA1 region during 2 min and the needles were left in place for an additional 60 s to allow for diffusion of solution away from the needle tip. DMSO and 3*α*- diol were injected 30-35 min and indomethacin 20-25 min before testing each day. The other procedure of injection was administered for rats that received two drugs or two doses of vehicle together. In this procedure 0.5 μL of DMSO plus 0.5 μL DMSO were injected in intact and sham group with 30 min interval, also 3 μg/ 0.5 μL of indomethacin plus 1 μg /0.5 μL of 3*α*- diol were injected with 25 min interval. Then the test began after a delay of 30 min each day.

*Behavioral assessment*

*Apparatus*

The Morris Water Maze (MWM) is one of the most frequently used laboratory tools in behavioral neuroscience (35) that relies on distal cues to navigate away from start locations around the perimeter of an open swimming area to locate a submerged escape platform (36, 37). The water maze is a black circular tank 140 cm in diameter and 60 cm in height. The tank was filled with water (21±1ºC) to a depth of 25 cm. The maze was located in a room containing many extra maze cues (*e.g*. bookshelves, refrigerator, and posters). The maze was divided geographically into four quadrants (Northeast (NE), Northwest (NW), Southeast (SE), Southwest (SW)) and starting positions (North (N), South (S), east (E),West (W)) that were equally spaced around the perimeter of the tank. A hidden circular platform (diameter 10 cm) was located in the center of the SW quadrant, submerged 1 cm below the surface of the water. A video camera above the water maze was connected to a video monitor and computer software (Ethovision XT, version 7), and was used to track the animals‘ path and to calculate the escape latency, traveled distance and swimming speed. 

*Procedure*

Each animal received four trials of one block per day in 4 consecutive days (11, 16). Each rat was placed in the water facing the wall of the tank at one of the four designated starting points and was allowed to swim and find the hidden platform located in SW quadrant of the maze on every trial. Starting points varied in a quasi-random fashion so that in each trial the animal started from each location only once and never started from the same place again. During each trial, each rat was given 90 s to find the hidden platform. If rat found the platform it was allowed to remain on it for 20 sec. If rat failed to find the platform within 90 sec, it was placed on platform for 20 sec. During the first 4 days the platform position remained constant. On day 5, the platform was elevated above the water surface and placed in SE quadrant. This assessed Visio-motor coordination toward a visible platform. All tests began at 8:00 am.

*Histology*

Following behavioral testing, animals were sacrificed by decapitation and brains were removed. For histological examination of cannulae and needle placement in CA1 region, 100 μm thick sections was taken, mounted on slides, stained with cresyl violet and the cannulae track was examined for each rat. Only those animals whose cannulae were exactly placed in the CA1 region were used for data analysis.

*Experiment 1*

The first experimental group consisting of intact and sham-operated rats received bilateral microinjection of 0.5 μL vehicle (DMSO or 0.5 μL DMSO+0.5 μL DMSO) into CA1 region of hippocampus. The aim of this experiment was to compare the effects of intrahippocampal vehicle injection with intact group of rats on MWM performance. A total of 27 rats were divided into three groups mentioned above. 

*Experiment 2*

The aim of this experiment was to determine the effect of intrahippocampal 3*α*-diol injection (one of the metabolites of testosterone), on MWM performance. A total of 40 rats were divided into four groups and received different doses of 3*α*- diol (0.2, 1, 3 and 6 μg/0.5 μL/side) 30-35 min before MWM test.

*Experiment 3*

The aim of this experiment was to determine the effect of intrahippocampal indomethacin injection (as a 3*α*-HSD inhibitor) on MWM performance. A total of 27 rats were divided into three groups and received different doses of indomethacin (1.5, 3 and 6 μg/0.5 μL/side) 20-25 min before MWM test. 

*Experiment 4*

The aim of this experiment was to determine the effect of intrahippocampal injection of indomethacin plus 3*α*- diol, on MWM performance. A total of 8 rats were received 3 μg/0.5 μL/side indomethacin + 1 μg/0.5 μL/side 3*α*- diol respectively, 30 min before test.

*Statistical analysis*

Statistical evaluation was done by Kolmogorov-semimov test at first, to examine the normal distribution. Data obtained over training days from hidden platform tests and in visible platform were analyzed by t-test for comparison between two groups and one- way analysis of variance (ANOVA) followed by Tukey,s test for multiple comparisons. All results have been shown as mean ± SEM. In all statistical comparisons, p < 0.05 was considered significant.

## Results

*Experiment 1: the effects of vehicles*

*Hidden platform trials (days 1-4)*

The results obtained from intact, vehicle (DMSO and DMSO+DMSO) indicate no significant difference in escape latency (*F*_2, 80_= 0.211, p = 0.810), traveled distance (*F*_2, 80_ =1.197, p = 0.307) and swim speed between groups (*F*_2, 80_=1.838, p = 0.166). These results indicated that injection of DMSO or injection DMSO+DMSO together had no effect on spatial learning and swimming ability.

*Visible platform trials (day 5)*

There was no significant difference in performance among intact, DMSO and DMSO+DMSO groups on the visible platform for latency (*F*_2, 19_=0.36, p = 0.965) or for traveled distance (*F*_2, 19_=0.347, p = 0.712).

*Experiment 2: the effect of 3α-diol*

*Hidden platform trials (days 1-4)*

**Figure 1:**
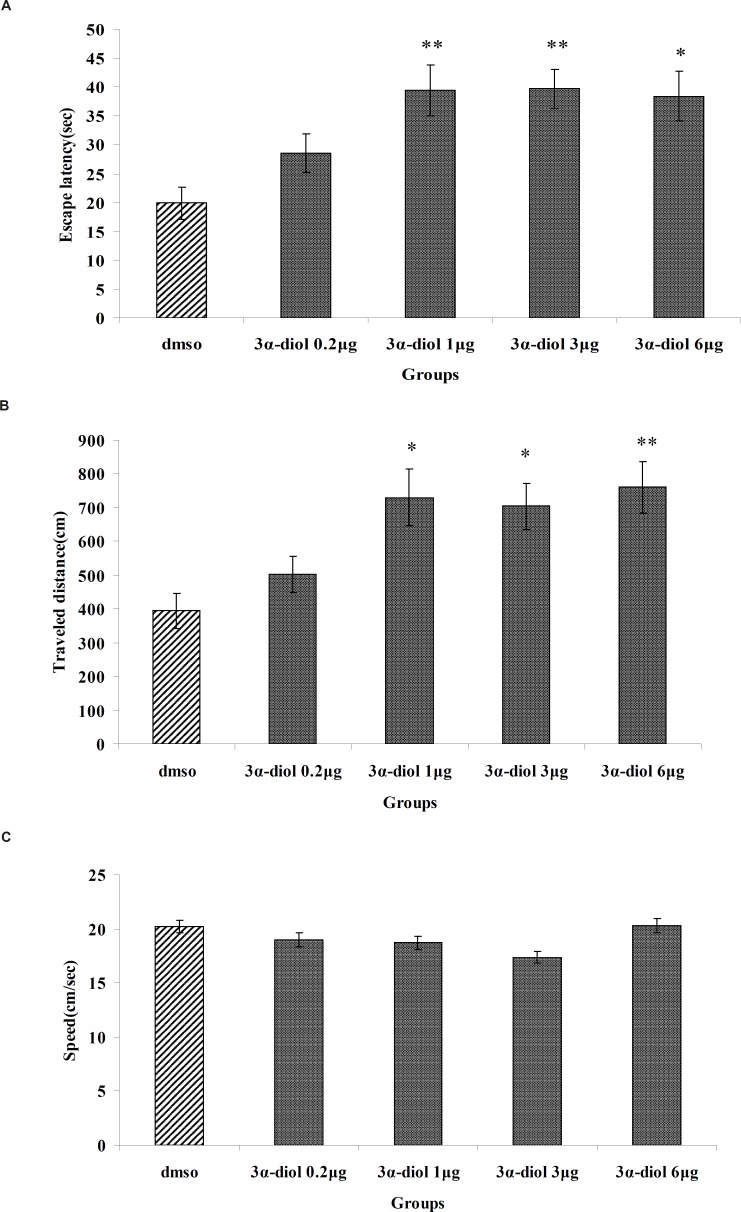
The effect of intra-CA1 administration of 3*α*-diol and DMSO on acquisition of spatial memory in MWM task. The columns represent the mean ± SEM. average escape latency (A) traveled distance (B) and swimming speed (C) across all training days (* p < 0.05 and ** p < 0.01 indicate significant difference vs. DMSO group).

Figure 1 show results obtained from the injection of 3*α*-diol or DMSO (control). A significant difference was generally found in escape latency (*F*_5, 166_= 5.713, p < 0.001) and traveled distance (*F*_5, 166_= 5.281, p < 0.001) between groups (fig. 1A and B). But no significant differences found in swimming speed (*F*_5, 166_= 1.825, p > 0.05) (Figure 1C). Post hoc multiple comparisons showed that 3*α*- diol at dose of 1, 3 and 6 μg significantly impaired acquisition of spatial learning compared to the control group. 

*Visible platform trials (day5)*

There was no significant difference of performance among the groups on visible platform day for escape latency (*F*_5, 41_=0.741, p = 0.597) or for traveled distance (*F*_5, 41_=1.173, p = 0.339).

*Experiment 3: the effect of indomethacin*

*Hidden platform trials (days 1-4)*

**Figure 2:**
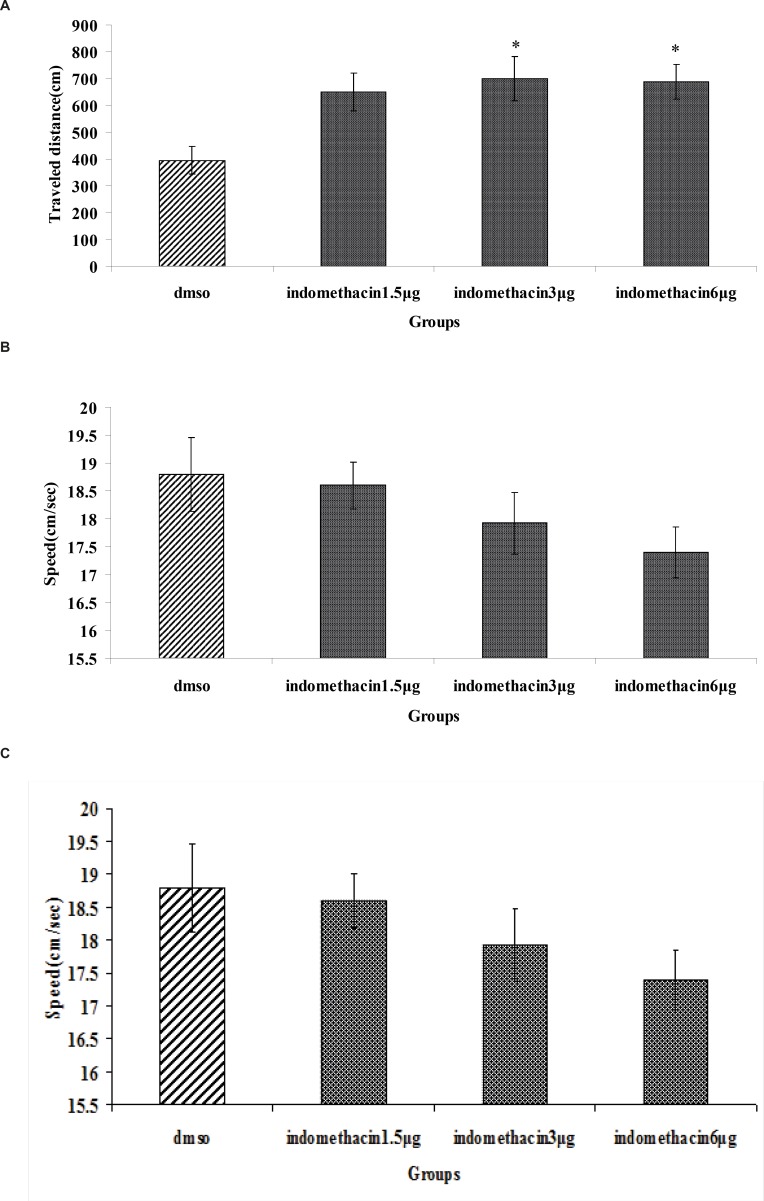
The effect of intra-CA1 administration of indomethacin and DMSO on acquisition of spatial memory in MWM task. The columns represent the mean ± S.E.M. average escape latency (A), traveled distance (B) and swimming speed (C) across all training days (* p < 0.05 and ** p < 0.01 indicate significant difference vs. DMSO group).

The effect of indomethacin or DMSO (control) is shown in Figure 2. A significant difference was generally found in escape latency (*F*_3, 112_=4.974, p = 0.003) and traveled distances (*F*_3, 112_=3.761, p = 0.013) between groups (Figure 2A and B). But no significant differences were found in swimming speed (*F*_3, 112_=1.530, p=0.211) (Figure 2C). Post hoc multiple comparisons showed that the rats treated with 3 and 6 μg indomethacin had significantly impaired acquisition of spatial learning compared to the control group. 

*Visible platform trials (day5)*

There was no significant difference of performance among the groups on visible platform day for escape latency (*F*_3, 28_=0.790, p = 0.510) or for traveled distance (*F*_3, 28_=2.625, p = 0.075).

*Experiment 4: The effect of indomethacin + 3α-diol*

*Hidden platform trials (days 1-4)*

**Figure 3:**
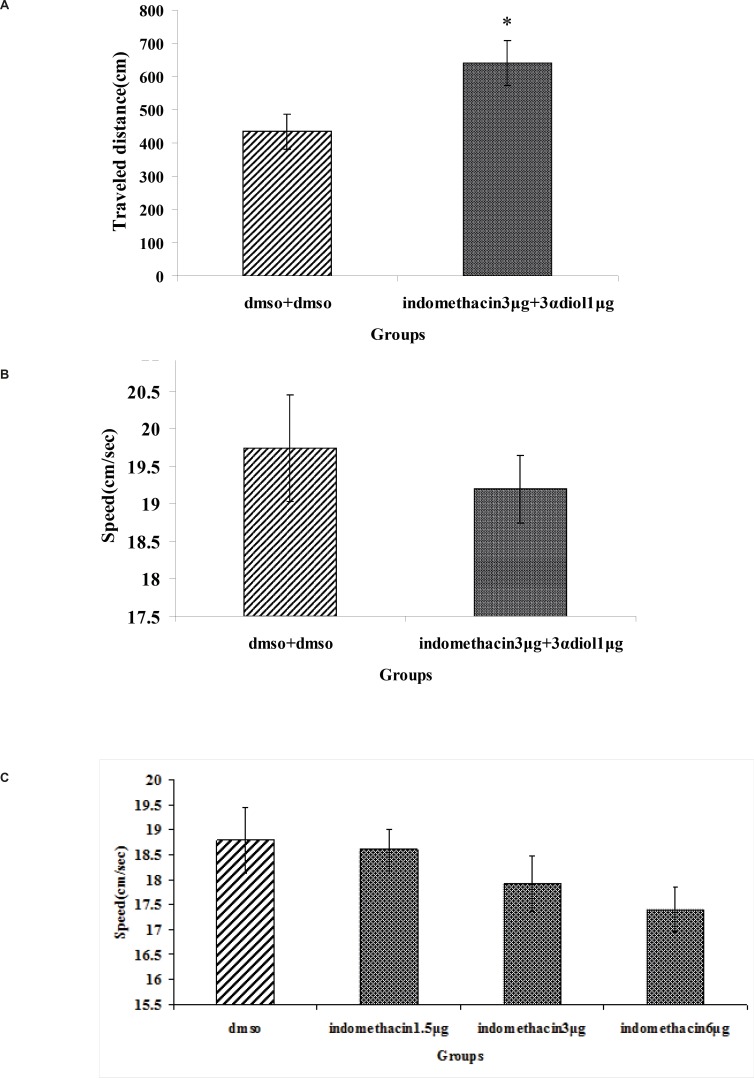
The effect of intra-CA1 administration of indomethacin + 3α- diol and DMSO + DMSO on acquisition of spatial memory in MWM task. The columns represent the mean ± SEM. average escape latency (A), traveled distance (B) and swimming speed (C) across all training days (* p < 0.05 indicate significant difference vs. DMSO + DMSO group).

A significant difference was generally found in escape latency (*T*_62_=4.65, p = 0.029) and traveled distance (*T*_62_=3.265, p = 0.02) (Figure 3A and B). But no significant differences were found in swimming speed (*T*_62_=10.885, p = 0.521) (Figure 3C). 

**Figure 4:**
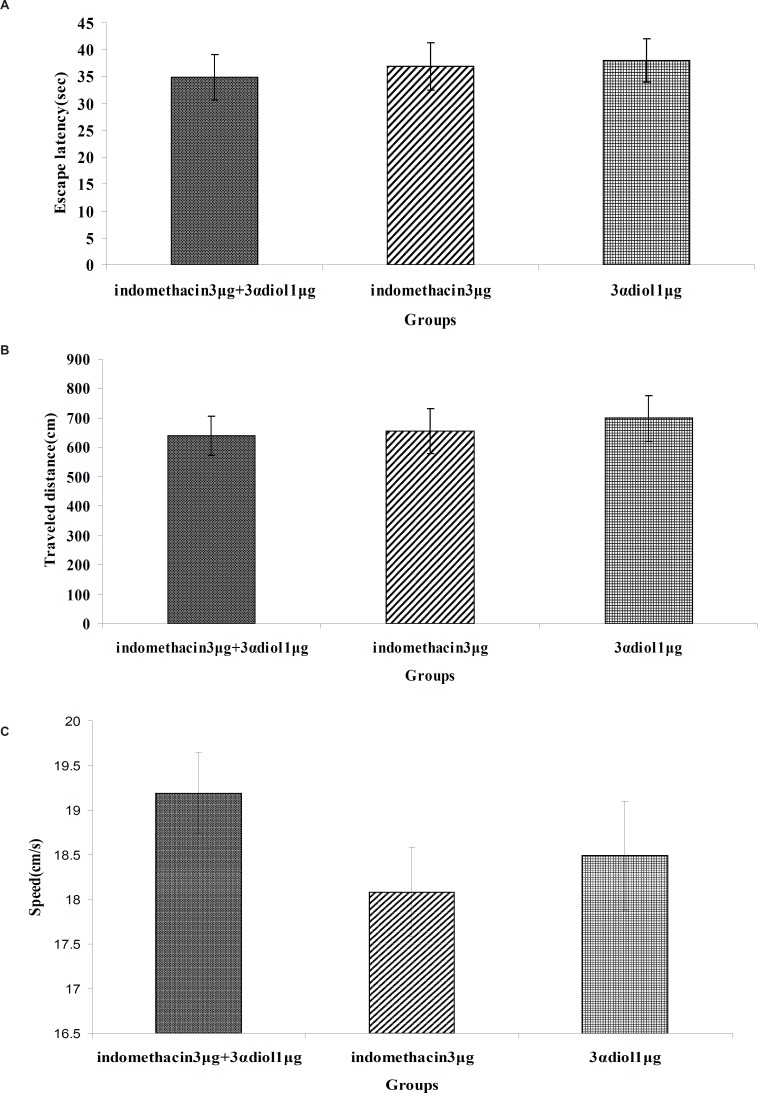
Comparison of the effect of intra-CA1 administration of indomethacin + 3*α*-diol, indomethacin and 3*α*-diol on acquisition of spatial memory in MWM task. The columns represent the mean ± SEM. average escape latency (A), traveled distance (B) and swimming speed (C) across all training days. There was no significant differences between the three groups

Fig. 4(A, B and C) revealed that there were no significant differences in escape latency(*F*_2,97_= 0.139, p = 0.870), traveled distance (*F*_2,97_= 0.170, p = 0.844) and swimming speed (*F*_2,97_=1.054, p = 0.353) between indomethacin 3 μg + 3*α*- diol 1 μg , indomethacin 3 μg and 3α- diol 1 μg group. These data indicated that presence of indomethacin can not inhibit impairing effect of 3*α*- diol on spatial memory. 

*Visible platform trials (day5)*

There was no significant difference of performance among the groups on visible platform day for escape latency (*F*_14_=2.912, p = 0.697) or for traveled distance (*F*_14_=3.204, p = 0.421).

## Discussion

Our experiments showed that there was not significant difference in spatial learning and memory among the intact rats and sham operated groups (DMSO and DMSO+DMSO). DMSO was used as a vehicle in other investigation with similar conditions (10, 11). In this experiment we saw that the double injection of vehicle (DMSO+DMSO) had no significant effect on learning and memory. This finding is consistent with some other reports (1, 5, 9, 10, 11, 16).

The results of experiment 2 indicated that intrahippocampal injection of 3*α*- diol in doses 1, 3 and 6 μg/0.5 μL impaired spatial learning in adult male rats in MWM task. 

Since there were no significant differences between the control and experimental groups on the 5^th^ day of training in visible platform, it can be inferred that the observed changes could not be attributed to alterations of non-mnemonic factors such as motivation, motor, or sensory processes induced by the treatments. 

This study, taken with other previous ones shows conflicting effects of androgens on cognition and suggests that cognitive-hormone interactions are quite complex. In several studies, androgens impaired spatial learning and memory (1, 5, 9, 10, 14-16) however, some other studies reported that androgens such as testosterone enhanced spatial memory (19-23). 

Androgens could exert these effects on memory through genomic and non-genomic pathways. The genomic pathway typically takes at least more than half an hour and involves long-term effects of androgens affecting gene expression via the intracellular androgen receptors. In addition to the classical genomic effects of steroids, many neurosteroids induce non-genomic effects by means of putative cell surface receptors that are manifested within in seconds to few minutes which can involve the modulation of neurotransmitter receptors and ion channels, ranging from activation of G-protein coupled membrane receptors or sex hormone- binding globulin receptors, stimulation of different protein kinases to direct modulation of voltage and gated ion- channels and transporters (5, 9, 11, 38-40).

Our previous study revealed that testosterone via both genomic and non-genomic pathways impairs long term memory (9). 

It has been demonstrated that neurons and glia express enzymes able to convert testosterone to estradiol and several 5α-reduced androgens such as dihydrotestosterone (DHT). Further metabolism of DHT results in the formation of 5*α*-androstane-3,17*β *–diol (3*α*-diol) (41), that is capable of eliciting estrogen receptor-dependent responses (42-44). There are several possible explanations for the impairing effect of 3*α*-diol on spatial memory. The first possibility is that the oxidative 3 hydroxy steroid dehydrogenases (HSDs) can convert 3α-diol back to DHT, leading to increased androgenic stimulation. Since, DHT has stronger androgenic effect than to 3*α*-diol (44), the obtained results may be related to the conversion of 3*α*-diol to DHT then to testosterone. In this way, androgen responsiveness is considered to depend on steroid transforming enzymes in brain (44).

The second possibility is based on this fact that in addition to the effect on estrogen receptor (ER), 3*α*-diol also affects on learning and memory through non-genomic pathway. One of the best-documented examples of non-genomic actions of steroids is the ability of these hormones to activate GABA_A_ receptors. Neurosteroids have been reported to modulate GABAergic function by increasing GABA_A_ receptor opening frequency and duration (6). Activation of the GABA_A_ receptor complex by such neurosteroids results in opening of its central Cl^- ^conducting pore, which leads to a hyperpolarization of the plasma membrane and inhibition of neuronal firing (40). The major groups of neuroactive steroids and their metabolites are progesterone, dehydrocorticosterone and some of their metabolites. 3*α*-diol acts like the analogous metabolites of progesterone and corticosterone, such as allopregnanolon, that enhances GABA-benzodiazepine regulated chloride channel function (42, 44, 45). In general, they mediate their actions not through classic steroid receptors, but through other mechanisms such as ligand-gated ion-channels including GABA_A_, glutamate or opioid receptors (40). On the other hand the biphasic effects of progesterone are consistent with a critical role for GABA-mediated responses. Progesterone, like DHT, is rapidly converted in the brain to 5*α*–reduced metabolites, some of which potentiate GABA action on the GABA-benzodiazepine-chloride channel complex (6). Therefore the effects of androgens such as 3α-diol could be mediated in much the same way as the rapid responses of progesterone, via enhancement of GABAergic neurotransmission.

Beside, some studies have shown that all hippocampal subregions are rich in GABA_A_ receptors and that some neurosteroids such as allopregnanolone can inhibit neural activity in the CA1 and dental gyrus areas of the hippocampus (3, 46, 47). Treatment with GABA_A_ receptor active substances such as, benzodiazepine, can inhibit learning and memory in humans and animals (3, 38). Acute treatment with neurosteroids that have GABA-modulatory effects impairs learning and memory. In contrast, steroids that act as GABAA receptor antagonists enhance learning and memory (48-50). 

The third possibility is based on this fact that there is an interaction between steroids and the serotonin system in the hippocampus. Some evidence showed that steroids such as estrogen and also progesterone metabolites such as alloprognanolone can affect spatial learning and memory via the serotonin system (19). Additionally, a direct interaction between the GABA and the serotonin systems in the hippocampus is proven, where serotoninergic neurons often end at inhibitory GABAergic interneurons (3, 51). Many studies have shown that excitation of serotoninergic neurotransmission impairs learning and memory, whereas, reduction of serotonin activity can improve these processes (51-54). Serotonin and GABA systems may therefore interact in the hippocampus; a region important for cognitive functions (3, 56) and 3α-diol, probably via this way induce impairment in learning and memory performance. 

Steroid hormones also modulate the memory processes perhaps by their relationship with other neurotransmitter system such as acetylcholine, dopamine, noradrenaline and glutamate, and by their relationship with the cerebral regions that participate in these phenomena (5, 10). Based on the evidences, testosterone and its metabolites, 3*α*-diol, can reduce acetylecholine release in the hippocampus, via positively modulationg hippocampal GABAergic interneurons that is shown to induce memory impairment (5, 57). Many studies have also shown that NMDA receptors are critical for synaptic plasticity and long term memory (LTM) (53, 58, 59). Testosterone by acting as non-selective sigma (σ) antagonist may produce a tonic damping of the function of sigma receptors and consequently a decrease in NMDA receptor function (5, 60). Therefore, these facts provide a reliable evidence for the explanation the effects of 3*α*-diol (as a one metabolite of testosterone) on spatial learning and memory.

The results of experiment 3 indicated that intrahippocampal injection of indomethacin at doses 3 and 6 μg/0.5 μL impaired spatial learning in adult male rats.

The results of experiment 4 showed that intrahippocampal injection of indomethacin 3 μg/0.5 μL +3α A-diol 1 μg/0.5 μL, impaired spatial learning and memory in adult male rats, similar to indomethacin and 3*α *A-diol. 

Our reason for using indomethacin come from the Frye *et al. *(2010) which mentioned that indomethacin can act as 3*α*-HSD inhibitor. Further blocking testosterone’s or DHT‘s metabolism to 3α-diol with indomethacin decreases cognitive performance and increases anxiety behavior of gonadally-intact and/or DHT-replaced rats (20, 28). In experiments 2 and 3 we assayed the effect of 3*α*-diol and indomethacin alone on learning and memory. Our finding shows that both 3*α*-diol and indomethacin impaired acquisition learning and memory performance. In experiment 4, we used indomethacin as 3*α*-HSD inhibitor to prevent the effect of endogenous 3*α*-diol on learning and memory and then studied the effect of exogenous 3*α*-diol on the acquisition stage of learning and memory. Our results show that exogenous 3*α*-diol has impairment effect on acquisition memory and indomethacin could not prevent the impairing effect of 3*α*-diol while indomethacin had impairment effects of its own. Beside, there is no significant difference between intra-CA1 administration of indomethacin + 3*α*-diol, indomethacin and 3α-diol on acquisition stage using one way ANOVA. It is possible that 3*α*-diol’s effects to impair cognitive performance cannot be influenced by indomethacin. On the other hand, indomethacin as a nonselective COX inhibitor (31-33), and can affect learning and memory. Thus, COX (cyclooxygenase) enzymes catalyze the first two committed steps in the biosynthesis of prostanoids. Several lines of evidence indicate a potential role for COX in the physiological mechanisms underlying memory function and indomethacin as COX inhibitors impair these mechanisms. For example, indomethacin as a COX inhibitors impairs passive avoidance memory in chicks and prevents the learning- induced increase in prostaglandin (PG) release, which occurs 2 h after training (33). 

In summary, it is concluded that intra CA1 administration of 3*α *diol and indomethacin could impair spatial learning and memory. Also intra hippocampal injection of indomethacin plus 3*α*-diol could not change spatial learning and memory impairment effect of indomethacin or 3*α*-diol in MWM task.
